# The influence of age and sex on speed–strength performance in children between 10 and 14 years of age

**DOI:** 10.3389/fphys.2023.1092874

**Published:** 2023-02-21

**Authors:** Konstantin Warneke, Carl Maximilian Wagner, Andreas Konrad, Björn Kadlubowski, Andre Sander, Klaus Wirth, Michael Keiner

**Affiliations:** ^1^ Department for Exercise, Sport and Health, Leuphana University, Lüneburg, Germany; ^2^ Department of Training and Exercise Science, German University of Health and Sport, Berlin, Germany; ^3^ Institute of Human Movement Science, Sport and Health, University of Graz, Graz, Austria; ^4^ University of Applied Sciences Wiener Neustadt, Wiener Neustadt, Austria

**Keywords:** triangle test, squat jump, countermovement jump, children, change of direction

## Abstract

**Introduction:** Speed-strength performance is important during human movements such as jumping, sprinting, and change of direction (COD) tasks, which are a substantial part of sports practice. Sex and age seem to influence performance output in young persons; however, few studies have focused on the influence of sex and age measured via standard protocols of performance diagnostics.

**Method:** Therefore, the aim of this study was to investigate the influence of age and sex on linear sprint (LS), COD sprint, countermovement jump (CMJ) height, squat-jump (SJ) height, and drop-jump (DJ) height performance in untrained children and adolescents via a cross-sectional analysis. This study comprised 141 untrained male and female participants 10–14 years of age.

**Results:** The results showed the influence of age in male participants on speed-strength performance, while in female participants, age did not significantly influence performance parameters. Moderate to high correlations between sprint and jump performance (*r* = 0.69–0.72), sprint and COD sprint performance (*r* = 0.58–0.72), and jump and COD sprint performance (*r* = 0.56–0.58) were found.

**Discussion:** Based on the data from this study, it appears that the growth phase of age 10–14 does not necessarily lead to improvements in athletic performance. To ensure holistic motor development, female subjects in particular should be provided with specific training interventions with a focus on strength and power.

## Introduction

Speed–strength performance, especially when utilizing a stretch-shortening cycle (SSC), is a key factor in explaining the efficiency of human movement and locomotion (Aura and Komi, 1986). During maturation from childhood to early adolescence, children become increasingly efficient in SSC performance, which is supposedly due to enhanced neuromuscular coordination and muscle mass (Jensen et al., 1994; Temfemo et al., 2009). The improvement of speed–strength performance is very important for enhancing jump, sprint, and change of direction (COD) performance. It is, therefore, an essential component in a wide range of sports, such as basketball (Ramirez-Campillo et al., 2021; Sugiyama et al., 2021), soccer (Altmann et al., 2019; Lohmann et al., 2022), handball (Pereira et al., 2018), and tennis (Jansen et al., 2021). In addition to sports performance, speed–strength performance has also been shown to be a predictor for functional capacity (Pijnappels et al., 2008).

With regard to the relationships between different explosive strength parameters, moderate correlations have been found between different COD sprints of 10–20 m, linear sprints (LS) (*r* = 0.4–0.6) (Suarez-Arrones et al., 2020; Hernández-Davo et al., 2021), and various jump performances (*r* = −0.4–0.68) (Castillo-Rodriguez et al., 2012; Hernández-Davo et al., 2021). Similarly, jump height and LS performance have shown moderate correlations (*r* = −0.43 to −0.59) (Suarez-Arrones et al., 2020).

However, there appear to be sex-specific similarities and differences in the correlation between COD sprint performance and jump, as well as sprint performance in men and women. Sekulic et al. (2013) and McFarland et al. (2016) reported correlations for male and female participants with regard to jump and COD sprint performance, with *r* = 0.12 in male participants and *r* = −0.22 in female participants. Similarly, correlations have been reported between squat jumps (SJs) and various COD tasks—*r* = 0.31–0.35 (*p* < 0.05) in males and *r* = 0.41–0.44 (*p* < 0.05) in females (Sekulic et al., 2013).

For female participants, moderate to strong correlations have been reported between LS (30 m) (pro-agility and *t*-test) and countermovement jump (CMJ) height (*r* = −0.502–0.751), and LS (30 m) and SJ height (*r* = −0.502 to −0.681). Meanwhile, in male participants, moderate correlations have been found between LS (10 and 30 m) and CMJ height (*r* = −0.476 and −0.570), and between LS (10 and 30 m) and SJ height (*r* = −0.443 and −0.553), respectively (McFarland et al., 2016).

In the context of maturation and growth, Malina and Bouchard (1991) reported that jump height doubled between 5 and 13 years of age. This is supported by further cross-sectional studies that have reported an increase in speed–strength performance with increasing age (Katic and Bala, 2012; Laffaye et al., 2016), which could possibly be attributed to improved neuromuscular coordination (Jensen et al., 1994; Temfemo et al., 2009). Other studies have referred to changes in anthropometric characteristics—such as body size and weight, leg length, and muscle volume and strength—as very important for performance enhancement during maturation (Temfemo et al., 2009; Focke et al., 2013). Accordingly, there appear to be sex-related differences in the development of jump performance in childhood and early adolescence, which are accompanied by higher increases in leg length and muscle mass in boys compared to girls (Malina and Bouchard, 1991).

Since most studies have investigated these traits in trained children or children within sports club environments, the influence of age and sex cannot be sufficiently investigated because, in this setting, increases in performance cannot be solely attributed to growth, as training age also increases with maturation. Accordingly, while most studies have reported a higher performance in older boys or girls, this may not only be exclusively attributable to age or sex but also to increased training experience. Consequently, to investigate the influence of age and sex more precisely, other potential performance-influencing parameters should be excluded, if possible. Therefore, to investigate the influence of age and sex on performance parameters and to exclude the influence of increased training experience, this study aimed to investigate the influence of age and sex on LS, COD sprint, CMJ height, SJ height, and drop jump (DJ) height performance in untrained children and adolescents 10–14 years of age. It was hypothesized that there would be increases in performance with increasing age from 10 to 14 and that male participants would have higher performances than female participants.

## Methods

The study was designed as a cross-sectional study. To answer the research question, tests were carried out on two test days. On test day 1, SJ, CMJ, and DJ tests were conducted, with a 15-min break between tests. On test day 2, the participants performed the LS test and then the COD sprint test.

### Participants

A total of 141 children were recruited from different school classes, with an average of approximately 3 h physical activity classes per week (70 males [12.1 ± 1.3 years; body height: 154.8 ± 11.4 cm; body mass: 47.7 ± 12.6 kg; body mass index [BMI]: 19.7 ± 3.5 [range 13.3–31.5] kg/m^2^] and 71 females [11.8 ± 1.3 years; body height: 152.2 ± 9.7 cm; body mass: 47.7 ± 13.3 kg; BMI: 20.0 ± 4.0 [range 13.8–35.2] kg/m^2^]). There were 22 10-year-old children, 32 11-year-olds, 39 12-year-olds, 27 13-year-olds, and 21 14-year-old children. All were interviewed using a questionnaire to determine their physical activity level. The activity questionnaire (similar to MoMo-AFB (Schmidt et al., 2016)) covered everyday activities, sports within and outside of organized clubs, and physical and sporting activities in the age-specific setting (school). For each of these activity areas, responses for duration and frequency were requested. In addition, for sports (within and outside of organized clubs), participation in competitions was also established. Children were excluded from the study if they performed a regular and structured training program in a sports club. Participants performing competitive sports or more than 3 h of sports per week were excluded. Similar questionnaires have reported a test–retest reliability of 0.66, an overall intraclass correlation coefficient (ICC) of 0.68, and validity (compared to an ActiGraph accelerometer) of *r* = 0.29 (Jekauc et al., 2013). All children were involved in a familiarization session 1 week before the testing, which was identical to the test day. The participants entered the test days without performing any physical exercise beforehand and could therefore said to have recovered.

All participants and their parents were informed about the experimental risks involved with the research and provided written informed consent for participation. The study was performed with human participants in accordance with the Declaration of Helsinki (version 2013) and was approved by the Ethics Committee of the German University of Sport and Health (DHGS-EK-2021-002).

### Design and procedures

The warm-up for the test days consisted of non-specific running at low to medium intensity (50–70% of maximum heart rate) for approximately 5 min. Coordination exercises, including running with lifted knees, heeling, and side stepping, were then performed for approximately 5 min. Subsequently, three acceleration runs covering approximately 30 m were performed with short walking breaks. Overall, the total warm-up time on each test day was 20 min.

### Triangle test

**FIGURE 1:**
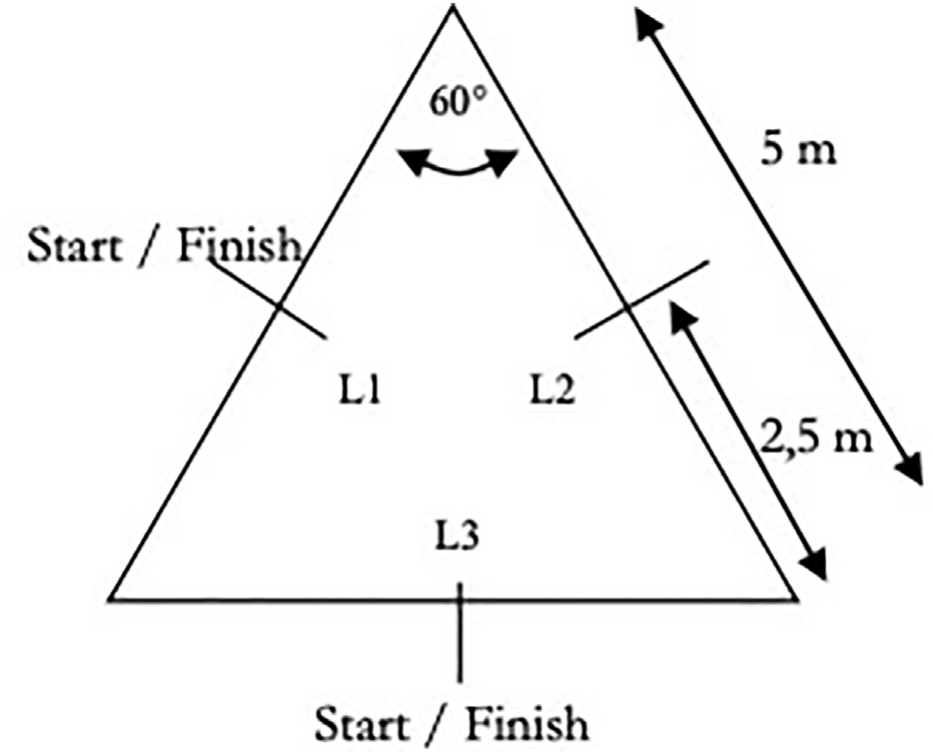
Illustration of the test procedure for the triangle test.

COD sprint performance was measured using the triangle test (TriT). A description of the test setup can be found in a previous study (Keiner et al., 2014) (Figure 1). Each subject individually performed three attempts for the TriT, with a 2-min break between each test. The total distance of the TriT was 10 m, with a change in direction after 2.5 m and again after 5 m. Since only one direction in turning can be tested *via* the TriT, each of the two directions was tested separately. If the pylons or hurdle bars were knocked down or touched during the COD testing, the test was repeated. The tests were separated by a 15-min break. The test–retest reliability, with an ICC of 0.89–0.94, was calculated in previous research (Kadlubowski et al., 2021).

### Linear sprint testing

LS performance was measured for distances of up to 30 m, with light barriers positioned every 5 m. Each participant performed three attempts, with a 2-min break in between. The time was measured for all the tests using a double-timing gate system (wk7 time watch, Ditzingen, Germany). The starting point was 0.75 m in front of the starting gate, to avoid early triggering by, for example, a hand movement or bent body position. The subject independently chose when the measurement began, according to the activation of the barriers. Thus, the reaction time was excluded from the measurement. The test–retest reliability reported ICC is 0.89 (Kadlubowski et al., 2021).

### Jump performance

Jump performance was measured using a contact mat (Refitronic, Schmitten, Germany). Each participant performed five trials for each jump. In the case of an invalid attempt, the measurement was repeated to exclude technique-related bias in the performance measures. The participant rested for 15 min between each jump test (CMJ, SJ, and DJ) and for 1 min between each attempt. The SJ test was initiated at a knee angle of 90°, without countermovement. Both the CMJ test and SJ test were performed with hands fixed at the hips, to avoid an arm swing movement. The DJ test was performed from a 16-cm height box. The participant was instructed to take a horizontal step from the box and to “fall” from the box onto the contact mat. After dropping onto the contact mat, the participant was asked to jump as high as possible by achieving a very short ground contact time (<250 ms) (CT). Thus, the participant’s heels were not allowed to touch the ground. Hands were fixed at the hips. From these data, the reactive strength index (RSI) was calculated (RSI = jump height (JH) in mm/CT in ms × 100) to quantify overall DJ performance. The test–retest reliability reported ICC for the jump tests is 0.85–0.93 (Keiner et al., 2018).

### Data analysis

The data were analyzed using SPSS 28.0 (IBM, Ehningen, DE, Germany). The significance level for all statistical tests was set at <0.05. Only the best value for each test was included for further analysis. The descriptive statistics for all measures are presented as the mean ± standard deviation (SD). Normal distribution was checked *via* a Kolmogorov–Smirnoff test. The best time for each test was used for further analysis. To assess the differences in performance between age groups, a mixed model ANOVA was performed considering sex and age. Effect sizes (ESs) for the global effect were calculated *via* the partial square of eta (η^2^). An ES of η^2^ ≥ 0.14 was classified as high, an ES of η^2^ ≥ 0.07 was classified as moderate, and an ES of η^2^ ≥ 0.01 was classified as small. For the pair-wise comparisons, a Scheffé test was used as the *post hoc* test and the ES was calculated *via* Hedges’ g. To determine significant differences in the correlation coefficients between subgroups (sex and age), the data were z-transformed according to the Fisher method. The difference of the two transformed values after standardization was assessed for significance as $z=\frac{z_{1}^{\prime }-z_{2}^{\prime }}{\sqrt{\frac{1}{n_{1}-3}+\frac{1}{n_{2}-3}}}$ . The Benjamini–Hochberg procedure was used to control the study-wise false discovery rate (FDR) at 0.05 (Ferreira and Zwinderman, 2006). Pearson correlation analysis was used to assess the relationship between the measured parameters, including the 95% confidence interval (CI). In general, an ES defined as g > 0.5 can be interpreted as large. Similarly, an ES ranging from 0.5 to 0.3 can be considered moderate, an ES from 0.3 to 0.1 small, and an ES of g < 0.1 can be considered trivial.

*Post hoc* power (1−ß) was calculated *via* G*Power 3.1.9.6 (University Düsseldorf, Düsseldorf, Germany) using the calculated ES and sample size of this investigation.

## Results

**FIGURE 2:**
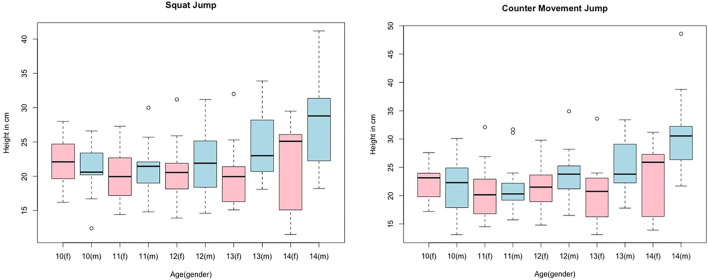
Box plots for jump height in SJ and CMJ dependent on age and sex; blue color represents males, and pink color represents females.

Normal distribution of data was confirmed by the Kolmogorov–Smirnoff test for most parameters. The descriptive statistics and the 95% CIs for the performance parameters and COD sprint performance are listed in Table 1. Descriptive statistics considering sex and age are provided graphically in Figure 1 and Figure 2, illustrated with box plots for CMJ, SJ, LS10m, and CODL10. The results of the ANOVA to investigate sex- and age-dependent effects on measured parameters are provided in Table 2.

**TABLE 1:** Descriptive statistics (mean ± SD) and confidence intervals for the performance parameters.

	Male (*n* = 70)	Female (*n* = 71)	Pooled (*n* = 141)
Fitness test	Mean ± SD [95% CI]	Mean ± SD [95%CI]	Mean ± SD [95%CI]
CODR (in s)	4.08 ± 0.45 [3.98–4.19]	4.37 ± 0.29 [4.30–4.44]	4.23 ± 0.4 [4.16–4.3]
CODL (in s)	4.03 ± 0.44 [3.92–4.13]	4.28 ± 0.29 [4.21–4.35]	4.15 ± 0.39 [4.09–4.22]
SJ (in cm)	23.2 ± 5.5 [21.9–24.5]	20.7 ± 4.6 [19.7–21.8]	21.9 ± 5.2 [21.1–22.8]
CMJ (in cm)	24.6 ± 6.1 [23.2–26.1]	21.3 ± 4.5 [20.2–22.3]	22.9 ± 5.6 [22–23.9]
DJjh (in cm)	19.3 ± 0.03 [17.92–20.98]	16.45 ± 5.51 [ 15.29–17.68]	17.87 ± 6.09 [16.84–18.90]
DJct (in ms)	229.77 ± 57.29 [ 215.93–243.37]	235.14 ± 38.67 [ 226.16–244.56]	232.46 ± 48.71 [225.21–240.25]
DJRSI	0.89 ± 0.38 [0.80–1.00]	0.72 ± 0.29 [0.66–0.79]	0.80 ± 0.35 [0.75–0.86]

COD, change of direction; R, right turn; L, left turn; SJ, squat jump; CMJ, countermovement jump; DJjh, drop jump height; DJct, drop jump contact time; DJRSI, drop jump reactive strength index.

**TABLE 2:** Results of mixed-model ANOVA to determine age- and group-specific differences.

Parameter	Age effect	Sex effect	Age × sex interaction
SJ	*p* = 0.037; ƞ^2^ = 0.074	*p* = 0.004; ƞ^2^ = 0.061	*p* = 0.039; ƞ^2^ = 0.073
	***✓***	***✓***	***✓***
CMJ	*p* = 0.002; ƞ^2^ = 0.12	*p* < 0.001; ƞ^2^ = 0.098	*p* = 0.025; ƞ^2^ = 0.081
	***✓***	***✓***	***✓***
DJjh	*p* = 0.027; ƞ^2^ = 0.08	*p* = 0.19; ƞ^2^ = 0.041	*p* = 0.221; ƞ^2^ = 0.42
	***✓***	***✕***	***✕***
DJRSI	*p* = 0.083; ƞ^2^ = 0.061	*p* = 0.008; ƞ^2^ = 0.52	*p* = 0.39; ƞ^2^ = 0.031
	***✕***	***✓***	***✕***
LS5m	*p* = 0.194; ƞ^2^ = 0.045	*p* < 0.001; ƞ^2^ = 0.118	*p* = 0.077; ƞ^2^ = 0.062
	***✕***	***✓***	***✕***
LS10 m	*p* = 0.072; ƞ^2^ = 0.063	*p* < 0.001; ƞ^2^ = 0.092	*p* = 0.089; ƞ^2^ = 0.059
	***✕***	***✓***	***✕***
LS20 m	*p* = 0.016; ƞ^2^ = 0.088	*p* = 0.001; ƞ^2^ = 0.078	*p* = 0.147; ƞ^2^ = 0.05
	***✓***	***✓***	***✕***
LS30 m	*p* = 0.013; ƞ^2^ = 0.09	*p* = 0.002; ƞ^2^ = 0.072	*p* = 0.159; ƞ^2^ = 0.049
	***✓***	***✓***	***✕***
CODL5	*p* = 0.630; ƞ^2^ = 0.019	*p* = 0.003; ƞ^2^ = 0.063	*p* = 0.233; ƞ^2^ = 0.041
	***✕***	***✓***	***✕***
CODL10	*p* = 0.50; ƞ^2^ = 0.025	*p* < 0.001; ƞ^2^ = 0.117	*p* = 0.257; ƞ^2^ = 0.039
	***✕***	***✓***	***✕***
CODR5	*p* = 0.477; ƞ^2^ = 0.026	*p* < 0.001; ƞ^2^ = 0.153	*p* = 0.52; ƞ^2^ = 0.024
	***✕***	***✓***	***✕***
CODR10	*p* = 0.563; ƞ^2^ = 0.022	*p* < 0.001; ƞ^2^ = 0.137	*p* = 0.30; ƞ^2^ = 0.036
	***✕***	***✓***	***✕***

The Scheffé test showed significant differences for SJ, height between ages 10 and 14 (*p* = 0.022; g = 3.2), 11 and 14 (*p* = 0.013; g = 1.39), and 12 and 14 (*p* = 0.035; g = 1.15) for boys, and for CMJ, height in the same age groups (*p* = 0.001–0.011; g = 1.33–1.61). However, in the female group, no significant differences could be found between the listed age groups (*p* = 0.29–0.79). Furthermore, the Scheffé test failed to show any significant difference between the other parameters (*p* = 0.057–0.999).

**FIGURE 3:**
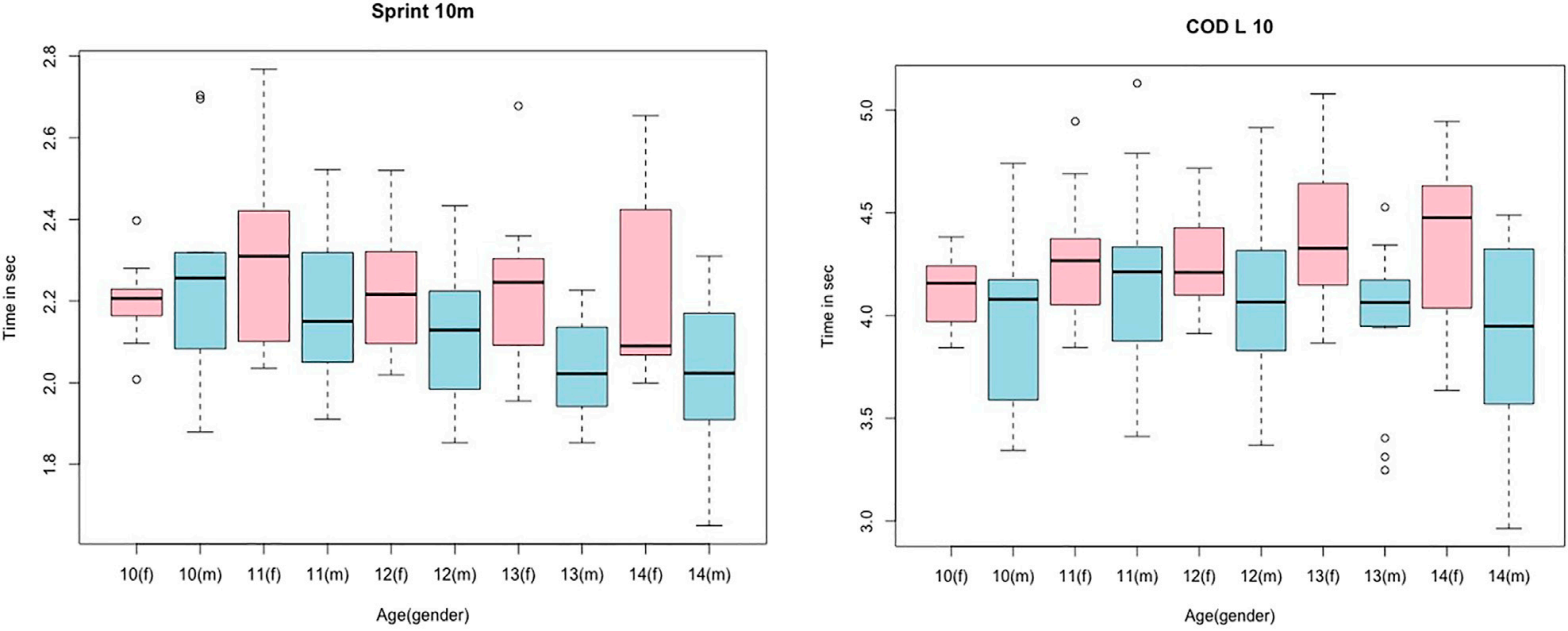
Box plots for jump height in LS10m and CODL10 dependent on age and sex; blue color represents males, and pink color represents females.

**FIGURE 4:**
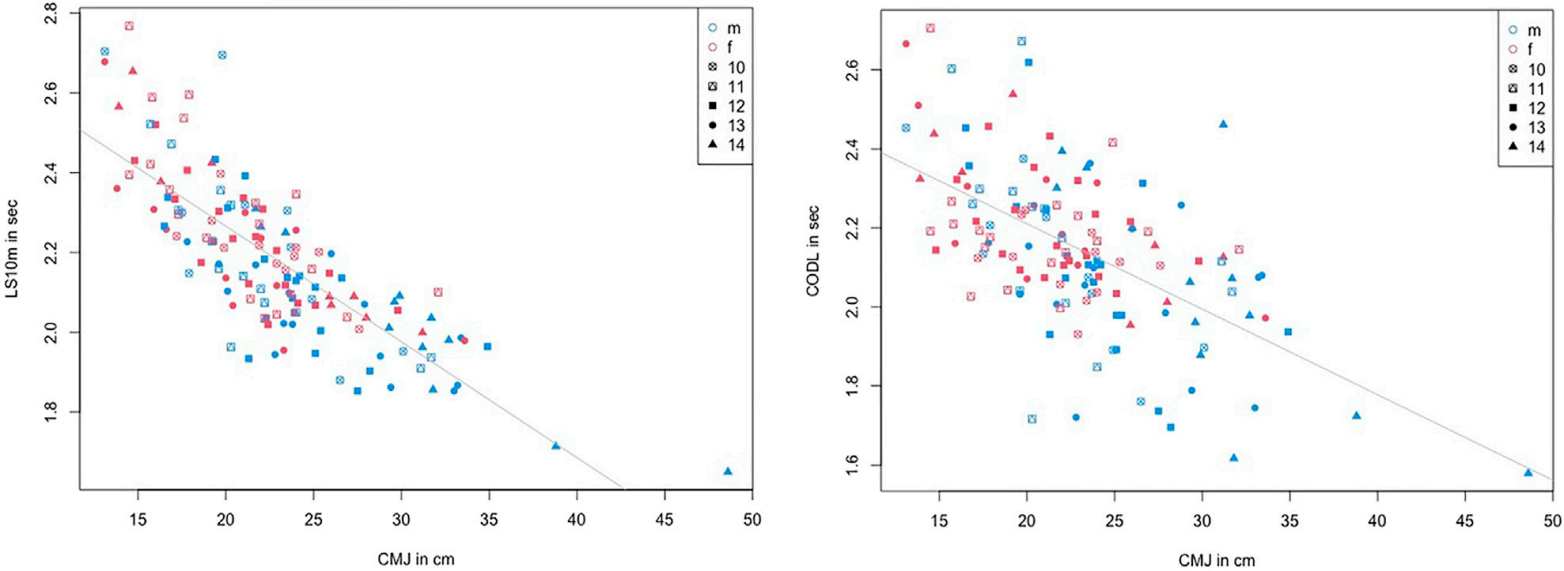
Correlation coefficients between linear sprint (10 m) and CMJ height, and COD sprint and CMJ height considering age and sex.

**FIGURE 5:**
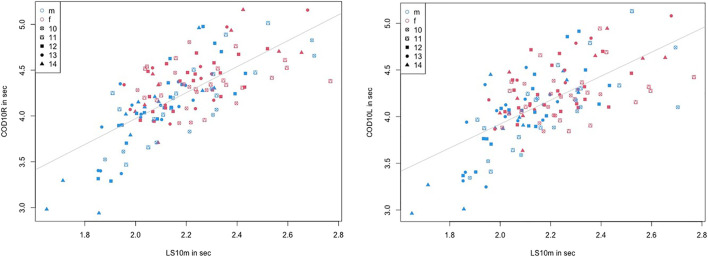
Correlation coefficients between COD right-turn sprint and linear sprint (10 m) and COD left-turn sprint and linear sprint (10 m) considering age and sex.

The correlations show no significant difference between different ages and sexes. Consequently, a correlation analysis could be performed which included all participants. The results are provided in Table 3, showing significant moderate to high correlations between jump height and LS, with *r* = 0.67–0.8, and low-to-moderate correlations between jump height and COD sprint performance, with *r* = 0.49–0.62. LS to COD sprint performance shows moderate to high significant correlations, with *r* = 0.58–0.73. However, no significant correlation can be determined for DJjh to DJct, with *r* = 0.14 and *p* = 0.11. Figure 2 and Figure 3 show the descriptive statistics considering age and sex. The correlation coefficients are illustrated in Figure 4 and Figure 5.

**TABLE 3:** Correlation matrix for the obtained parameters.

	SJ	CMJ	DJ (height)	DJ (contact time)	DJ index	LS5m	LS10m	LS20m	LS30m
CMJ	0.90 [9.86–0.93] *p* < 0.001								
DJ jh	0.66 [0.56–0.74] *p* < 0.001	0.70 [0.61–0.78] *p* < 0.001							
DJ ct	−0.22 [−0.58–0.60] *p* = 0.008	−0.24 [−0.39–0.80] *p* = 0.004	−0.14 [−0.30– 0.30] *p* = 0.11						
DJ RSI	0.632 [0.52–0.72] *p* < 0.001	0.68 [0.58–0.76] *p* < 0.001	0.86 [0.81–0.9] *p* < 0.001	−0.56 [−0.66–0.43] *p* < 0.001					
LS5m	−0.69 [−0.77–0.60] *p* < 0.001	−0.74 [−0.81–0.65] *p* < 0.001	−0.61 [−0.7–0.49] *p* < 0.001	0.26 [0.1–0.41] *p* = 0.002	−0.60 [−0.69 to −0.47] *p* < 0.001				
LS10m	−0.74 [−0.80–0.65] *p* < 0.001	−0.80 [−0.85 to −0.73] *p* < 0.001	−0.67 [−0.75–0.57] *p* < 0.001	0.37 [0.22–0.50] *p* < 0.001	−0.69 [−0.77 to −0.59] *p* < 0.001	0.94 [0.92–0.96] *p* < 0.001			
LS20m	−0.74 [−0.80 to −0.65] *p* < 0.001	−0.80 [−0.85 to −0.73] *p* < 0.001	−0.68 [−0.76 to −0.58] *p* < 0.001	0.40 [0.25–0.53] *p* < 0.001	−0.71 [−0.78 to −0.62] *p* < 0.001	0.88 [0.84–0.91] *p* < 0.001	0.98 [0.98–0.99] *p* < 0.001		
LS30m	−0.72 [−0.79 to −0.63] *p* < 0.001	−0.78 [−0.84 to −0.71] *p* < 0.001	−0.68 [−0.76 to −0.58] *p* < 0.001	0.40 [0.25–0.53] *p* < 0.001	−0.71 [−0.78 to −0.62] *p* < 0.001	0.85 [0.79–0.89] *p* < 0.01	0.96 [0.95–0.97] *p* < 0.001	0.99 [0.99–1.0] *p* < 0.001	
CODL5	−0.56 [−0.68 to −0.53] *p* < 0.001	−0.59 [–0.69 to −0.47] *p* < 0.001	−0.52 [0.63 to −0.39] *p* < 0.001	0.33 [0.17–0.47] *p* < 0.001	−0.6 [−0.69 to −0.48] *p* < 0.001	0.58 [0.46–0.68] *p* < 0.001	0.66 [0.56–0.75] *p* < 0.001	0.68 [0.58–0.76] *p* < 0.001	0.68 [0.57–0.76] *p* < 0.001
CODL10	−0.58 [−0.68 to −0.46] *p* < 0.001	−0.60 [−0.70 to −0.48] *p* < 0.001	−0.49 [−0.61 to −0.36] *p* < 0.001	0.29 [0.13–0.44] *p* < 0.001	−0.57 [−0.67 to −0.45] *p* < 0.001	0.62 [0.50–0.71] *p* < 0.001	0.66 [0.56–0.75] *p* < 0.001	0.67 [0.56–0.75] *p* < 0.001	0.66 [0.56–0.74] *p* < 0.001
CODR5	−0.56 [−0.66 to −0.43] *p* < 0.001	−0.62 [−0.71 to −0.50] *p* < 0.001	−0.54 [−0.65 to −0.41] *p* < 0.001	0.26 [0.10–0.41] *p* = 0.002	−0.58 [0.68 to −0.45] *p* < 0.001	0.65 [0.54–0.73] *p* < 0.001	0.72 [0.62–0.78] *p* < 0.001	0.73 [0.64–0.80] *p* < 0.001	0.73 [0.64–0.80] *p* < 0.001
CODR10	−0.57 [−0.67 to −0.44] *p* < 0.001	−0.62 [−0.72 to −0.51] *p* < 0.001	−0.55 [−0.65 to −0.42] *p* < 0.001	0.29 [0.13–0.44] *p* < 0.001	−0.60 [–0.7 to −0.48] *p* < 0.001	0.65 [0.54–0.73] *p* < 0.001	0.71 [0.62–0.78] *p* < 0.001	0.73 [0.64–0.80] *p* < 0.001	0.72 [0.64–0.80] *p* < 0.001

SJ, squat jump; CMJ, countermovement jump; DJjh, drop jump height; DJct, drop jump contact time; DJRSI, drop jump reactive strength index; LS, linear sprint, 5 = 5 m, 10 = 10 m, 20 = 20 m, and 30 = 30 m; COD, change of direction; R, right-turn; L, left-turn.

The results of the mixed model ANOVA are provided in Table 2. *Post hoc* power [1−β err prob] analysis resulted in a power of 82–99%.

## Discussion

The aim of this study was to investigate the influence of age and sex on LS, COD sprint, SJ height, CMJ height, and DJ height performance in children and adolescents 10–14 years of age. In addition, the influence of aging on the correlations of the performances was analyzed. Moderate to high correlations between jump height and LS performance (*r* = 0.67–0.8) and between LS and COD sprint performance (*r* = 0.58–0.73), and low to moderate correlations between jump height and COD sprint performance (*r* = 0.49–0.62) were found. Similarly, previous studies have reported a wide range of correlations, which can possibly be attributed to the high specificity of explosive strength requirements in different sports. Popowczak et al. (2019) reported correlations of *r* = 0.3–0.6 between jump performance and different COD testing, while Vescovi and McGuigan (2008) tested 213 athletes from high school and college in LS and jump performance, showing correlation coefficients of *r* = –0.66 to −0.79. Moreover, Pereira et al. (2018) reported correlations of *r* = 0.34–0.84 between speed–power performance and COD sprint performance.

According to Katic et al. (2012), there are significant differences in explosive strength parameters when considering age and sex for many measured parameters. In particular, in jump performance (SJ and CMJ), their results showed significant age and sex effects with a significant age × sex interaction. In sprint performance, there were also significant age and sex effects; however, no significant interaction effects could be obtained. Interestingly, the statistics indicated significant sex effects for all parameters, except DJjh. In general, their results corroborate the findings of Catley and Tomkinson (2013) and Tomkinson et al. (2018), showing significantly higher performance values in boys and older participants compared to younger participants, from 85,347 test values and 2,779,165 Eurofit performance test values from 30 countries. In addition, these studies also showed the greater influence of age on performance development in teenage boys compared to teenage girls. Similarly, Katic and Bala, 2012) reported higher performance values for explosive and agility performance in older participants (13–14 years old) compared to younger groups (10–12). Vescovi et al. (2011) showed a similar development pattern in female athletes by investigating their performance in LS, CMJ height, and two agility tests in 414 female soccer players of different age groups (12–21 years old). Accordingly, the authors reported higher performances in CMJ height and agility score in the older participants and suggested that their results could indicate that improvements due to high-intensity short-duration exercise do not occur until approximately 16 years of age in females. Similarly, Loturco et al. (2020) showed increased LS velocity over all age groups in 128 soccer players from U15 to seniors, but COD speed did not show changes in the younger ages and decreased with higher age. However, as explosive strength performance can be assumed to depend on the training level (Ostojic et al., 2010; Freitas et al., 2019a), sport specificity (Freitas et al., 2022; Loturco et al., 2022), and position profile (Freitas et al., 2019b), it is difficult to attribute higher values in performance exclusively to maturation processes since most studies were performed with participants from sports teams so that the higher training levels might bias these observations. However, no age-related differences could be obtained for the COD measurements. This could possibly be attributed to increased coordinative difficulty in COD testing. This might also explain why this study could not find any age-related differences in COD sprint performance as an increased coordinative difficulty supposedly requires specific training to enhance performance.

Various authors have indicated the significant influence of strength capacity on sprint and jump performance (Freitas et al., 2019a; Emmonds et al., 2019). Accordingly, Radnor et al. (2021) reported significantly higher jump performances in more mature boys and attributed these differences to increased muscle thickness compared to younger boys.

Therefore, it can be hypothesized that there is a significant influence of age on strength in boys (Parpa and Michaelides, 2022), and that, consequently, increases in measured performance values with enhanced age may be attributed to a higher relative maximum strength level due to pubescence, whereas, in female soccer players, a decrease in relative strength with increased age can be observed (Emmonds et al., 2020). There would appear to be sex-related factors that influence explosive strength performance. Freitas et al. (2021) measured agility and explosive strength in elite male and female rugby players and reported that male athletes showed higher performance in all tested parameters (DJ from 45-cm box, horizontal single and triple jumps, 40-m LS, pro agility, L-drill, zig-zag COD test, and 1RM in the squat). Men almost certainly demonstrated significantly higher performances than women in all the speed-power assessments and COD tasks (ES ranging from 0.61 to 2.09; *p* < 0.05) (Freitas et al., 2021). These results are in accordance with Pereira et al. (2018), who reported higher performances in jumping, sprinting, and COD performance in male compared to female elite handball players. In children and adolescents, Papaiakovou et al. (2009) reported significantly higher running speed and COD sprint speed (Meylan et al., 2014) in boys 7–18 years old. Since Emmonds et al. (2020) indicated that the growth of girls is often accompanied by a decrease in relative strength—with the potential to influence sports-related performance parameters such as sprinting and jumping—strength capacity training in girls can be hypothesized as being more important than previously assumed.

Furthermore, considering the results of Radnor et al. (2021), muscle mass might also influence performance output in speed–strength performance, and the male muscle cross-sectional area can typically be assumed to be higher than that in females (Bishop et al., 1989; Stock et al., 2020; Elsharkasi et al., 2022).

Differences in maximum strength and the muscle cross-sectional area can be partially attributed to significant differences in sexual hormones (i.e., testosterone) (Handelsman et al., 2018), which partially explains the differences in the muscle cross-sectional area and maximum strength. Handelsman (2017) and Tønnessen et al. (2015) pointed out that a performance gap opens between boys and girls at age 12–13, which is in accordance with the results of this study, accompanied by a rise of circulating testosterone in boys during puberty.

In addition, Katic et al. (2012) and Drid et al. (2013) reported significant differences in cognitive motor functioning in prepubertal and pubertal children. These differences might have an impact on explosive strength, jump, and sprint performance and might be influenced by maturation and growth. Furthermore, Llyod et al. (2012) suggested that higher supra-spinal feed forward input and shorter latency stretch reflexes influence SSC due to maturation in children between 9 and 14 years of age (*p* < 0.01).

The results of the present study showed significant age-related differences in untrained children between male and female participants aged 10–14. Although increased age led to increased jump and sprint performance in boys, the current study could not observe this progression in untrained female participants. However, there was no significant effect for the magnitude of correlation between these test items.

## Limitations

This paper provides data about the correlations between jump and sprint performance in untrained participants, and the results are (partially) in accordance with previous research in trained children and adolescents. However, it seems beyond debate that strength capacity affects speed–strength ability and is therefore of importance for jump and sprint performance (Styles et al., 2016; Suchomel et al., 2016; Warneke et al., 2022). Consequently, a maximum strength measurement was missing in this study. However, untrained children were included, and consequently, it was not possible (i.e., due to the lack of the technique) to perform a maximum strength test in, for example, squats to calculate the correlation between strength capacity and performance. Furthermore, limitations can be seen in measuring the LS5m times because of the high percentage of errors attributed to variations when starting. To counteract this problem, participants had to start 0.75 m before the first light barrier, and a double light barrier measurement system was used. Based on the study design (i.e., *ad hoc* samples), *a priori* sample size estimation *via* G*Power was not feasible. Therefore, the differences must be interpreted with caution. Still, based on the *post hoc* G*Power analysis, the sample size was sufficient to allow a solid evaluation (power for age differences [1–β err prob]: 82–99%). In addition, a cross-sectional design was adopted in this study; therefore, these are not dependent samples. In principle, reference should be made to the reliability and validity of the questionnaire for the assessment of physical activity, which should be evaluated with a certain degree of caution, based on the correlation coefficients. Nevertheless, the questionnaire appears to be comparable with other questionnaires in terms of validity and reliability (Jekauc et al., 2013). In addition, we did not test the maturity status and body composition of the analyzed samples; this information would have allowed even more detailed conclusions to be drawn from the data. Nevertheless, the data appear to be valuable against the background of a limited number of studies, although the differences between the age groups should be viewed with caution with regard to longitudinal evaluation. Lastly, some of the investigated parameters, such as CMJ height and LS5m, were not normally distributed, which could enhance the possibility of type 1 and 2 errors.

## Practical applications

The data presented here represent a first step to obtaining normative performance data for children in maturation and growth, and consequently, the derived practical application remains hypothetical since no training intervention took place. However, becoming older (with developmental changes) does not necessarily seem to lead to improvements in athletic performance, based on the data obtained in this study. Sports participation is an important mediator in terms of performance improvement (Keiner et al., 2013). However, against the background of physical inactivity (pandemic-related, among other things), remaining active is also important in health prevention. The large number of inactive and overweight children and adolescents must give an occasion to introduce children to activities or sports. Furthermore, being overweight and inactive can lead to secondary complications. Therefore, parents and coaches should help inactive children to participate in sports. Coaches should take these findings into consideration when constructing training programs for young people of different training levels. They should design training programs with difficulties adapted to the training level of the children to boost the performance of the training and encourage inactive and overweight children to participate. This does not mean that inactive children should only be exposed to a low to moderate training stimulus. After they have shown enthusiasm for the sport, this group also needs a progressive increase of training stimulus to obtain training adaptations.

## Data Availability

The raw data supporting the conclusions of this article will be made available by the authors, without undue reservation.
